# Energy-Efficient Monitoring of Fine Particulate Matter with Tiny Aerosol Conditioner

**DOI:** 10.3390/s22051950

**Published:** 2022-03-02

**Authors:** Sung Hoon Baek

**Affiliations:** 1 Gonggam Sensors Co., Ltd., Daejeon 34129, Korea; shun@ggsensors.com or shbaek@jwu.ac.kr; Tel.: +82-43-830-1193; 2 Department of Computer Engineering, Jungwon University, Geosan-gun 28024, Korea

**Keywords:** particulate matter, PM, monitor, sensor, tiny aerosol conditioner, light scattering, TAC, TAM

## Abstract

Fine particulate matter (PM) is associated with an increased risk of respiratory and cardiovascular diseases. Fine PM absorbs water molecules at high relative humidity, and then their size grows. Such hygroscopic growth causes a large error when monitoring PM concentrations. To lower the relative humidity, monitors use an indirect heating device, which is large and consumes large amounts of power. The problem with conventional particle separators is that their efficiency depends on temperature and humidity, and their traditional structure, which lets air flow downward. As such, this paper addresses these problems and presents a PM monitor with a new type of dryer that is free from these problems. The proposed monitor requires less energy and has an efficient dehumidifier and a new structure in which air flows upward. The presented experiments were conducted to compare the proposed device with a reference monitor managed by a governmental institute, and to evaluate the effect of the dehumidifier, the relative precision of the proposed devices, and the correlation with the reference monitor. The experimental results showed that the proposed monitor satisfies the U.S. EPA indicators for class III monitors.

## 1. Introduction

Four main methods are used for measuring the concentration of particulate matter (PM): gravimetric monitor, beta-radiation attenuation monitor (BAM), tapered element oscillating microbalance (TEOM), and optical particle monitor (OPM).

Gravimetric monitors measure the two weights of the clean filter and the dirty filter where PM accumulates with a predefined air volume for one day and obtains the weight of PM by subtracting the two measured weights.

BAMs indirectly measure the weight of PM using the beta radiation attenuation. BAMs collect atmospheric air during one hour and measure the amount of attenuation of beta radiation that passes through the filter accumulated with PM. The time resolution of BAM is typically an hour. A BAM model, such as Met One BAM 1020, requires a one-hour cycle, which consists of 4∼8 min for beta measurement, 42 min for air sampling, and 2 min for tape and nozzle movements [1]. Some of the BAM models can provide an output every minute [2].

TEOM uses a small vibrating glass tube whose oscillation frequency changes when aerosol particles are deposited on it [3,4]. A TEOM model provides an average output every ten seconds [5].

OPMs can measure PM concentrations in seconds. They are often called light scattering monitors. Due to their small size and low cost, they can be applied in various fields by combining them with Internet of Things (IoT) technology [6,7,8]. OPMs measure the intensity of scattered light when a laser passes through an aerosol. Light intensity that is detected on a photodiode depends on the aerosol size; thus, the monitor can count the number of particles and measure their diameter. The weight of aggregated PM is translated using a coefficient from the measured particle size distributions.

Fine dust particulate matter that is composed of ammonium nitrate, ammonium sulfate, and inorganic salts has a hygroscopic property, which is the ability to absorb water molecules at high humidity to increase their size by three to four times [9,10].

The minimum level of relative humidity at which aerosols absorb water molecules is called the deliquescence point, which depends on the composition of the particles. Single salts have lower deliquescence points than multicomponent aerosols [11,12].

PM monitors that do not control relative humidity produce different results depending on the relative humidity and the composition of aerosols [10,13,14,15]. Hygroscopic growth results in OPMs measuring the grown size of particles and BAMs measuring the weight of water. Therefore, PM monitors require a dryer that returns aerosols back to their initial moisture-free state.

### 1.1. Problems with Conventional Devices

**Figure 1:**
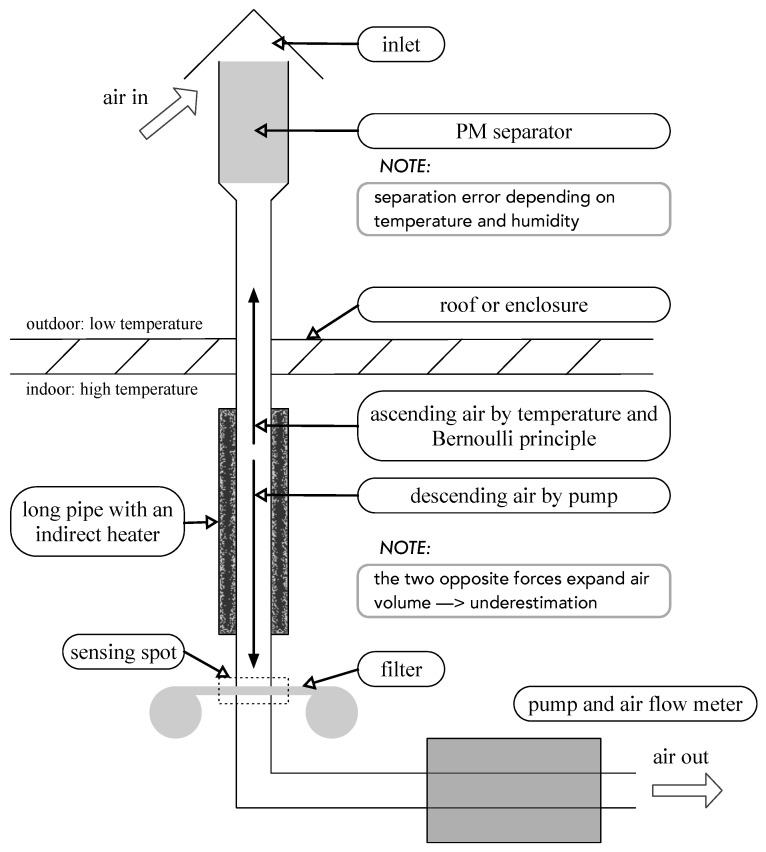
Diagram of a traditional monitor.

Figure 1 is a diagram of a traditional PM monitoring device. Air flows into the inlet located at the uppermost position of the device. Large particles in air are filtered out by an impactor or a cyclone separator. The liquid water in hygroscopic particles evaporates while flowing along the long warm pipe with a heater.

PM deposits on the glass fiber filter. The weight of aggregated PM on the filter is measured hourly with a BAM or daily with the gravimetric method. The air flows downward with a constant airflow rate produced by a pump. The amount of airflow is controlled by an airflow meter. In most cases, 16.5 L of air flows per minute [1,16].

BAMs have a tape-shaped glass fiber filter inside the sensing spot. Some sophisticated OPMs (GRIMM EDM, Teledyne T640) have a similar structure to that of BAMs except for their sensing spot, which is composed of a laser and a photodiode. Teledyne’s T640, an OPM certified by the U.S. EPA, has a 109.2 cm long pipe-type aerosol sample conditioner to dry deliquescent aerosols up to 35 to 40% of relative humidity.

A BAM, Thermo Fisher’s Model 5014i, maintains its 91.4 cm pipe at a specific temperature using a direct heating method. A heater is attached around a metal pipe through which the air sample passes. The heater heats the pipe, and the heated pipe lowers the relative humidity of the air sample [17].

The conventional PM monitors exhibit the following various problems:

1. **Dryer placed after separator:** Because the dryer is located behind the particle separator, particle size can increase due to their hygroscopic growth and, in turn, might be removed from the separator [18]. This is why the traditional system underestimates PM concentrations under high-humidity conditions.
2. **High energy consumption:** A large volume of air is required to aggregate enough PM in the filter. For example, BAM 1020 of Met One Instruments Inc. (Grants Pass, OR, USA) requires a nominal flow rate of 16.7 L/min. A pump model, BX-121, used in BAM 1020, requires 520 W [1]. A heater model, BX-830, used in BAM 1020, consumes 175 W to dehumidify aerosols [1]. Additional power is required to maintain the proper temperature of the shelter enclosing the monitor. A shelter heater, BX-902B, and a shelter air conditioner, BX-904, consume 500 and 1172 W, respectively [1].
3. **Downward airflow against buoyancy:** In winter, air inside the pipe has an upward buoyant force because of the temperature difference between the outside and inside of the shelter. The heat from the dryer further increases this force. The pump makes the air flow downward. The combination of the upward and downward force lowers the air pressure inside the pipe and expands the air volume. This leads to underestimation of PM concentrations by erroneously measuring the air volume.
4. **Inefficient separator at high temperatures:** The temperature dependency of an impactor causes errors when used as a particle separator. High temperature makes its impaction plate unstable, which in turn lowers the impactor performance [19,20]. High temperature increases the re-bounce of the particles that are captured on the plate and decreases the viscosity of the oil on the plate. Additionally, the efficiency of the cyclone separator decreases at high temperatures [21,22]. Hence, during summer, the traditional system overestimates PM concentrations.
5. **Frequent maintenance:** Many parts require frequent cleaning. Separators should be cleaned weekly or bi-weekly. The nozzle requires monthly cleaning.
6. **Wind-sensitive intake pipe**: Wind decreases the air pressure inside the pipe. From Bernoulli’s principle, air pressure inside the pipe depends on the square of the wind velocity *v* and the height of the pipe *h*. The air pressure *P* is defined as:
  (1) $$P+\frac{1}{2}\rho v^{2}+\rho gh=constant,$$
  where ρ is the air density. The change in the air pressure, ΔP, is:
  (2) $$\Delta P=-\frac{1}{2}\rho v^{2}$$
  Low air pressure expands the air volume according to Boyle’s law. On that account, strong wind likely affects monitor performance, resulting in underestimation of PM concentrations.

Most monitors experience the problems mentioned above, but some monitors have the technology to resolve some of them. An OPM model, GRIMM EDM 180, uses a long Nafion dryer instead of a heater, thereby extracting isothermal humidity. Thermo Scientific Model 5030 is a hybrid monitor of BAM and OPM. It regulates humidity levels using a heating system that is linked to a relative humidity sensor [23].

### 1.2. Contribution

The proposed method compensates for a large number of the limitations of the traditional methods. The presented method, a tiny aerosol conditioner inside an air monitor (TAM), has been applied to a product model, GGS727, which received Grade 1 certification for OPM by the Korean Ministry of Environment in April 2021.

The regulation of the Korean certification is similar to class III FEM of the U.S. EPA, but the former needs 336 hourly average samples at one site and the latter requires a total of 115 daily data from four sites.

The advantages of TAM over the traditional method are as follows:

1. **A small dryer placed before logical separator:** The proposed device includes a tiny aerosol conditioner (TAC) that dehumidifies air with low power. The device logically separates the particle size by measuring the size of the dust at the sensor without a physical separator. Both PM measurement and logical separation are performed after dehumidification. Hence, during both separation and measurement steps, hygroscopic growth is not possible.
2. **Low energy consumption:** The proposed monitor, TAM, requires up to 2.9 W of power for dehumidification. Generating airflow requires 0.11 W. All electronic circuits including the airflow fan consume 1.8 W. TAM requires a total of 3.7 W. A traditional system consumes a total of 715 W, which consists of a 175 W heater, a 520 W pump, and a 20 W main device.
3. **Upward airflow following the direction of buoyant force:** In contrast to conventional devices, the proposed device makes air flow upward from the bottom with low air pressure using low electric ventilation power (0.11 W). Since the direction of the airflow is the same as that of buoyant force, this results in the expansion of air volume being negligible and lowers energy requirements.
4. **Logical separator independent of temperature:** The proposed system uses the light scattering method, so particle size separation is unaffected by temperature. This is because the diameter of each particle is electronically measured by the Mie scattering principle.
5. **Minimal maintenance:** Periodic cleaning is not required because dust cannot accumulate on device parts with a perfectly vertical structure.

## 2. Design and Implementation

### 2.1. Dehumidifier with Upward Airflow: TAC

Inorganic salts account for most of PM’s composition. These salts can increase in size due to hygroscopic growth at a relative humidity of 35%. A deliquescence point at which the solid aerosol is liquefied is observed at a relative humidity of around 67% [24].

Hygroscopic growth results in inaccurate OPM measurement of the particle size. As such, this paper presents TAM, an OPM that includes a tiny aerosol conditioner (TAC) that dehumidifies aerosols at a relative humidity of 35%, which is lower than the deliquescence point.

**Figure 2:**
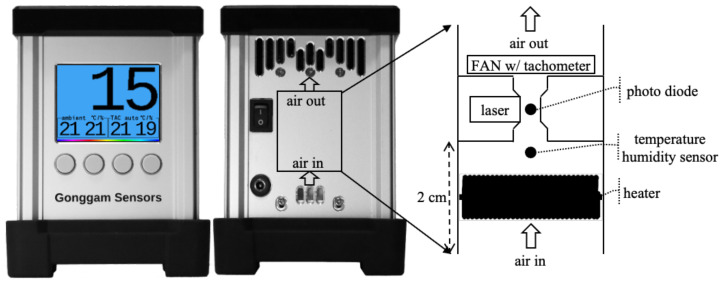
Back side of the proposed monitor and the internal structure of the tiny aerosol conditioner with the pneumatic flow (dehumidifier with upward airflow).

Figure 2 demonstrates the front and back view of the proposed device and the internal TAC structure. TAC has a compact, low-power, direct heater. Air flows upwards, and the inlet and outlet are exposed in the same direction to prevent wind interference.

Air enters the inlet. The dense coil heater has a large contact area, allowing it to quickly raise the air temperature. This coil also serves to protect the sensors from insects and large dust particles such as dandelion spores. A laser and a photodiode are located in the sensing spot. The cross-sectional area of the heater (288 mm${}^{2}$) is wider than that of the sensing spot (9 mm${}^{2}$), so that the air velocity in the heater unit is less than that in the sensing spot. As a result, the air can be heated efficiently.

The airflow rate is approximately 2 L/min, but the controller does not ensure a constant flow but compensates the final measurement result with the instant airflow rate that is measured by a tachometer.

Traditional methods such as BAMs and gravimetric monitors use a mechanical particle separator that is placed before the dehumidifier. This traditional placement causes error in the separation stage when dealing with hygroscopic aerosols. A small aerosol that is supposed to pass through the separator can grow larger and will eventually be filtered out by the separator due to hygroscopic growth. In addition, the high temperature lowers the efficiency of this separator [19].

TAM experiences no hygroscopic error in particle separation because it deals with the particle size after dehumidification. TAM, as an OPM, optically measures the size of each particle by Mie scattering; thus, it does not require a mechanical particle separator.

The proposed device, TAM, has a dryer called TAC. The air entering from the inlet is heated by the coil heater of TAC to lower the relative humidity (RH), and the water absorbed by aerosols evaporates.

A temperature/RH sensor in TAC measures the temperature and RH of the heated air, and the main circuit board controls the current of the heating coil to keep the relative humidity of the air constant by proportional-integral-derivative (PID) control. Air with constant RH passes through the sensing spot that measures the PM concentration.

At temperatures above 40 °C, inorganic salts can be formed through atmospheric chemical reactions and some semivolatile substances disappear. [24,25,26,27]. The temperature of the heater in TAC is controlled so that it does not exceed 40 °C.

### 2.2. Upward Airflow and High Reliability

For the case of traditional monitoring methods, the inlet is located at the top, and the pump at the bottom lowers the air pressure, making the air flow downward. As such, the air pressure at the pump depends on the wind speed, length of the pipe, and the difference between the ambient temperature and heated air temperature.

According to Bernoulli’s equation (Equation (1)), the longer the pipe, the lower the air pressure inside the pipe; therefore, the pump needs more energy. In addition, depending on the wind speed passing through the inlet, the dynamic change in air pressure inside the pipe changes the airflow rate. A device that manually controls the strength of the pump may have a huge error depending on the wind speed. Other monitors that automatically control the pump to keep the airflow rate constant also have error when they cannot quickly adapt to the rapidly changing wind speed.

In winter, a strong buoyancy occurs in the pipe due to a large difference between the ambient temperature and the inside temperature heated by a dryer and a shelter heater. For the air to flow against this buoyancy, the pump needs a large amount of energy.

Both the high temperature and low air pressure of the pump cause the air volume to expand. Thermometers and barometers are needed on both the outside and in the pump to compensate for this expansion of the air volume.

However, the proposed method was designed so that both the inlet and outlet are exposed to the outside in the same direction so that wind does not affect the air pressure inside the device. Since the direction of airflow is equal to that of the buoyant force that is caused by the heater, no additional energy is required for buoyancy. The power of a small fan with a speedometer is just 0.11 W, which is enough to generate the required airflow. The fan’s speedometer measures the airflow rate. The firmware of the main control board compensates for the expansion of the air volume due to the heater from TAC by using both the ambient thermometer and the thermometer in TAC.

The underestimation of PM concentrations owing to the expansion of the air volume by heat can be compensated by the following equation according to Boyle’s law:

(3) $$\mathrm{compensated}\;\mathrm{PM}\;\mathrm{concentration}=‘\mathrm{uncompensated}\;\mathrm{PM}\;\mathrm{concentration}’\times \frac{T_{t}+273}{T_{a}+273},$$

where $T_{t}$ and $T_{a}$ are the temperature of TAC and the ambient temperature in degrees Celsius, respectively.

The dust deposition reduces the durability of the monitor or requires periodic maintenance. As shown in Figure 2, the perfectly vertical structure prevents dust from accumulating on a surface. In particular, the sensors are arranged in an orthogonal direction on the vertical axis so that dust does not accumulate on the sensors. This structure ensures high durability and does not require periodic maintenance.

The dense coil heater protects the sensors against large particles and insects. Large particles blocked by the coil heater fall by gravity. Large particles of dust hardly rise towards the coil heater and fall. Since the inner walls of the chamber are composed of a material with a very small coefficient of friction, dust cannot easily be deposited in the heater chamber.

In the steady state, the heater chamber is heated close to the temperature of the coil heater. The temperature difference between the inner wall of the heater chamber and the coil heater is around 2 °C. Most of the air is evenly heated by the dense coil heater and chamber walls. Airflow in the same direction as buoyancy prevents recirculation.

### 2.3. Block Diagram

**Figure 3:**
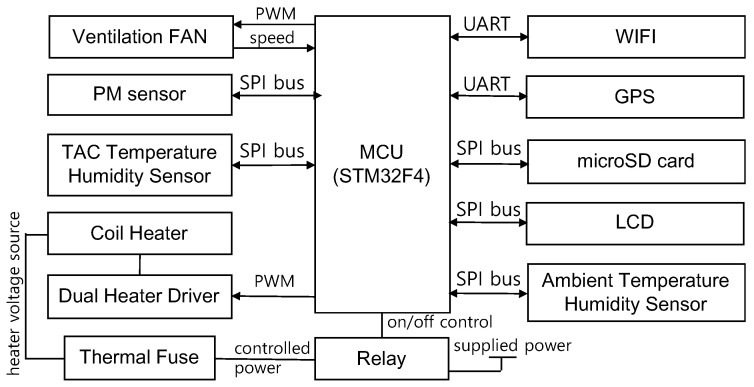
Block diagram of the proposed device.

Figure 3 demonstrates a block diagram of the proposed control board. MCU is a system on chip with a 32-bit CPU, ARM Cortex M4, which has many interfaces for the serial peripheral interface (SPI), pulse width modulation (PWM), universal asynchronous receiver transmitter (UART), and general input output.

The ambient temperature/humidity sensor is installed to compensate for the increased air volume caused by the heater. The ventilation fan removes heat generated from the board. This enables accurate measurement of the ambient temperature. The control board has several UART interfaces for a WiFi module and a GPS module.

The coil heater inside TAC is driven by a power metal oxide semiconductor field effect transistor (MOSFET) that controls the power of the heater in a PWM manner. TAC has various methods to prevent fire from breaking out. When the temperature inside the device is uncontrollably high, the firmware cuts off the heater power using a relay. If the TAC temperature is still uncontrollable due to an unknown fault, the power wire is cut by a thermal fuse.

TAC consists of a coil heater, a temperature/RH sensor, and an enclosure, which is composed of a material that provides insulation, anti-static, and electrical isolation functions.

The microSD card can store measured data for at least 60 years. The WiFi module transmits data to a data server. The GPS provides both the measurement location and the precise measurement time. The GPS time is also used as a time token constraint method [28] for the data server to protect against cloning attacks.

## 3. Experimental Results

The performance of the proposed device, TAM, was evaluated in various ways. First, we analyzed the relative precision between devices to evaluate the errors between multiple TAM devices, as described in Section 3.1. Second, the effect of TAC is analyzed in Section 3.2. Third, in Section 3.3, TAM is compared with a BAM device that is managed by a Korean governmental institute, the National Institute of Environmental Research (NIER).

This experiment evaluated TAM during autumn and winter in 2021 with the open data from a branch of NIER that is in Daejeon, South Korea. NIER used Met One’s BAM1020 model as the BAM.

### 3.1. Relative Precision

This section evaluates the relative precision between multiple TAM devices. The experimental results were obtained from three TAM devices (candidate methods) at the headquarters of the Korea Testing Laboratory in Jinju from 25 September to 9 November 2021.

The relative precision for OPMs is defined by the U.S. EPA [29]. According to the U.S. EPA, an estimate for the relative precision of the candidate method (test device) on day *j*, $CP_{j}$, is defined by

(4) $$CP_{j}=\frac{1}{{\bar{C}}_{j}}\sqrt{\frac{\sum_{i=1}^{m}C_{i,j}^{2}-\frac{1}{m}{\left(\sum_{i=1}^{m}C_{i,j}\right)}^{2}}{m-1}}\times 100\%,$$

where $C_{i,j}$ is the daily average concentration of the *i*th device on test day *j*, ${\bar{C}}_{j}$ is the mean concentration of the all devices on day *j*, and *m* is the number of candidate methods, which must be at least three.

The candidate method relative precision, CP, is defined by the root mean square of Equation (4) as presented below:

(5) $$CP=\sqrt{\frac{1}{J}\sum_{j=1}^{J}{\left(CP_{j}\right)}^{2}},$$

where *J* is the total number of valid measurement days, which must be 23 for each test campaign.

**Figure 4:**
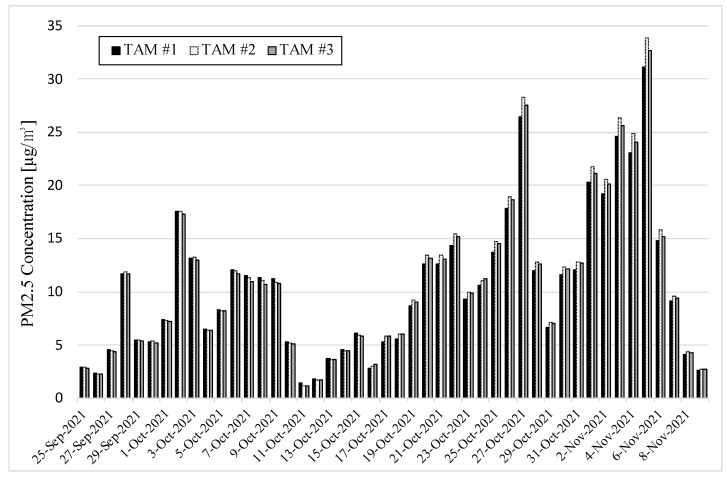
Comparison of three devices.

Figure 4 shows the daily average values of ${\mathrm{PM}}_{2.5}$ concentration obtained from the three devices for 46 days. The U.S. EPA regulation states that the relative precision CP must be 15% or less for class III monitors.

Class III, class II, and class I correspond to OPMs, BAMs, and gravimetric monitors, respectively.

From this experimental result, the relative precision of the proposed device was 3.25%. This proved that the error between the TAM devices is small, meaning that it shows high repeatability.

### 3.2. Assessment of TAC

This experiment shows the comparison of three monitors: a TAM device with TAC-on, a TAM device with TAC-off, and the BAM device used by NIER as a reference monitor. TAC-off represents the same hardware components as TAC-on but with no electric power for the heater. The measurement results of this BAM device are published every hour on the AirKorea website. TAM delivers hourly average data for comparison with the hourly data of the reference monitor.

This experiment was performed from 1–30 November 2021. Two TAM devices, one with the dryer and the other without, were placed on the roof of a building located within 20 m of the NIER building.

Here, each measurement datum for a TAM device with TAC-on, a TAM device with TAC-off, and the BAM device of NIER is referred to as ‘TAC-on’, ‘TAC-off’, and ‘NIER’, respectively.

**Figure 5:**
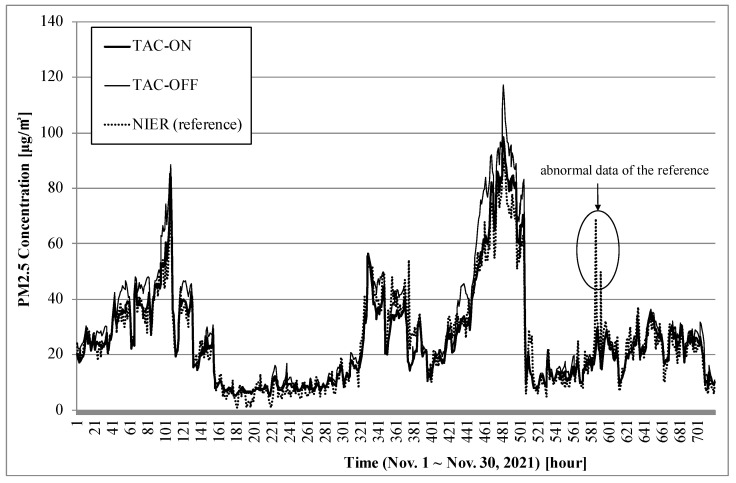
PM concentrations of two TAM devices with TAC-on and TAC-off from 1–30 November 2021.

Figure 5 illustrates the ${\mathrm{PM}}_{2.5}$ concentrations of TAC-on, TAC-off, and NIER for 30 days in November 2021. TAC-on showed similar results to NIER, but TAC-off did not.

**Figure 6:**
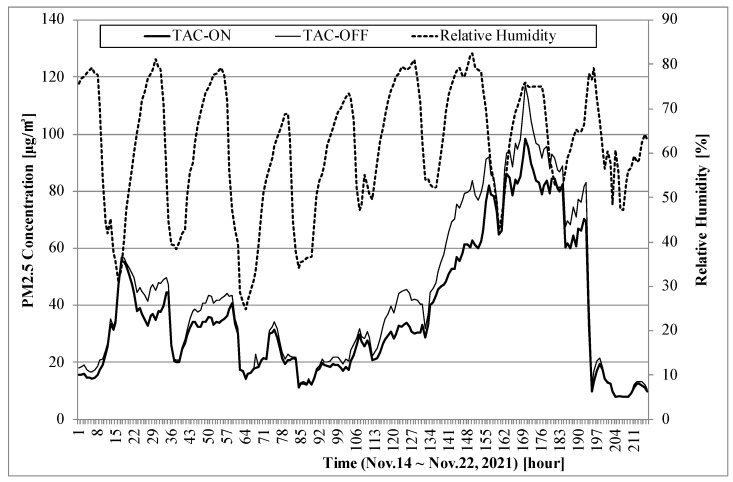
PM concentrations of two TAM devices with TAC-on and TAC-off from 14–22 November 2021.

Figure 6 shows the ${\mathrm{PM}}_{2.5}$ concentration and the relative humidity of the two TAM units from 14–22 November 2021. This result demonstrates that TAC-off overestimated the PM concentration measured during night when the relative humidity was high; also, these two TAM devices showed similar results during daytime when the relative humidity was low. The difference was particularly wide when a high PM concentration occurred. In November, TAC-off overestimated by 13% on average and by 44% at the maximum.

TAC requires lower energy for dehumidification. Even if the heater is turned off, the heat generated from the main control board is transferred to TAC; accordingly, the relative humidity decreases by 9% on average. If humidity had not dropped due to the heat of the circuit board, TAM with TAC-off would have shown a larger error.

**Figure 7:**
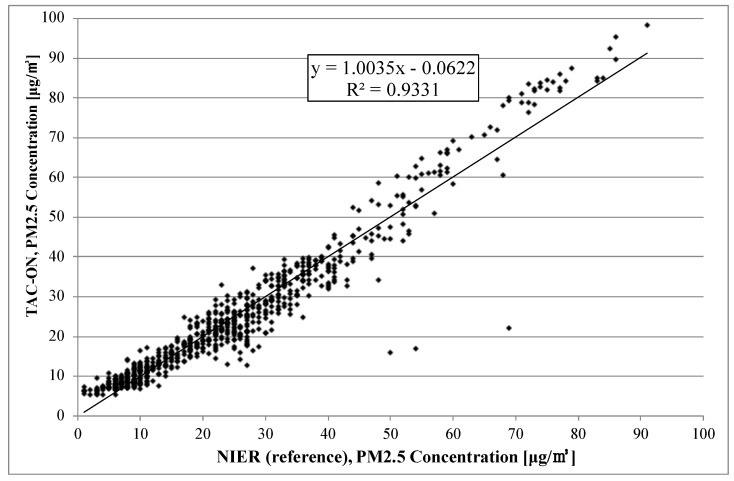
Correlation between TAM with TAC-on and the reference method (BAM) from 1–30 November 2021.

**Figure 8:**
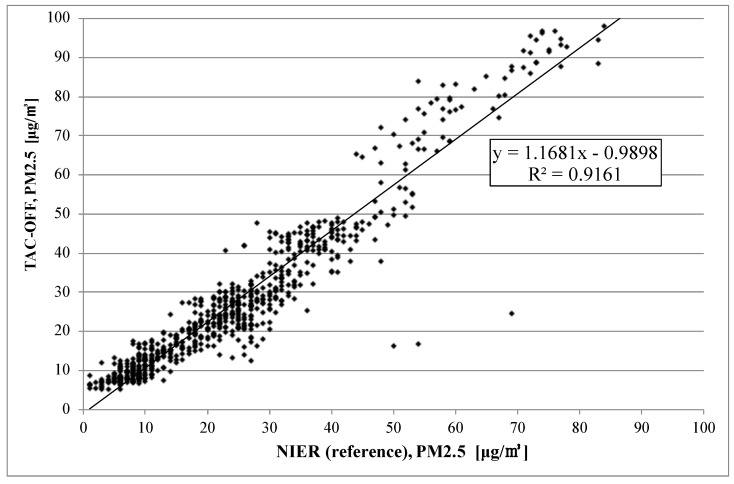
Correlation between TAM with TAC-off and the reference method (BAM) from 1–30 November 2021.

Figure 7 and Figure 8 show the correlation between the reference method (NIER) and the two TAMS: TAC is turned on in Figure 7 and off in Figure 8. For the former case, the slope of the trend line was around 1.004, and $R^{2}$ is 0.933. For the case with TAC turned off, the slope was 1.168, which is worse than that of TAC-on. However, $R^{2}$ was smaller, with a value of 0.916. The coefficient of determination $R^{2}$ determines the correlation between two measurement sets. The closer the $R^{2}$ to one, the higher the similarity of these two measurements.

Some abnormal data were included in the case of the reference monitor, as shown in Figure 5. This worked as a factor in decreasing the value of the coefficient of determination, but it is not the error for TAM.

**Figure 9:**
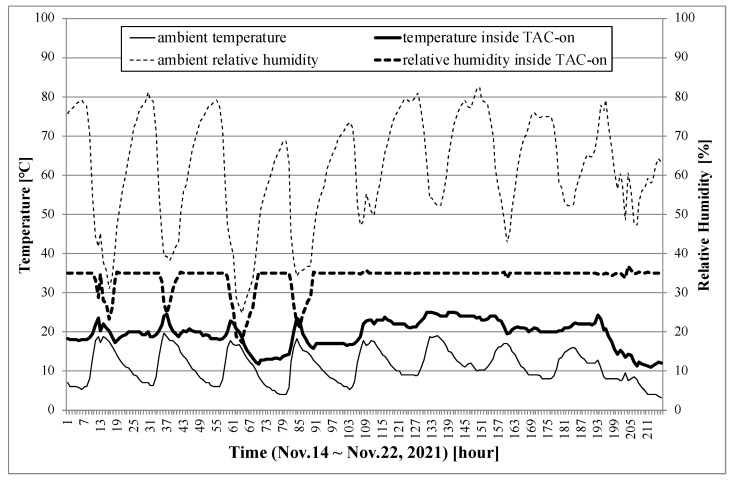
Comparison of temperature and relative humidity between inside and outside of TAC-on from 14–22 November 2021.

Figure 9 compares the temperature and relative humidity (RH) within TAC-on with the ambient temperature and RH from 14–22 November 2021. The RH inside TAC was always maintained below 35% by the automatic controller. The ambient RH decreased during the day and increased at night. The ambient RH was less than 35% around noon during November 14 to 17 (1 to 96 h). At this time, the heater of TAC was turned off and the RH of TAC was similar to the ambient RH, but slightly lower than ambient RH due to the heat of the circuit board. During November 18 to 22 (97 to 216 h), the ambient RH humidity was always above 35%, even during the day, and the heater of TAC continued to operate day and night. The maximum temperature difference between the inside and outside of TAC was 13.5 °C. At this time, the PM concentration compensation according to Equation (3) was +4.8%.

### 3.3. Slope, Intercept, and Correlation Coefficient

Existing mechanical particle separators perform best at −2 °C [19]. As such, TAM, as a target, and a BAM, as a reference, were compared during winter from 26 January to 9 March 2021. A BAM device was used as the monitor for NIER. TAM was placed on the same site as the NIER monitor. The targets were not calibrated with the reference for this test campaign.

**Figure 10:**
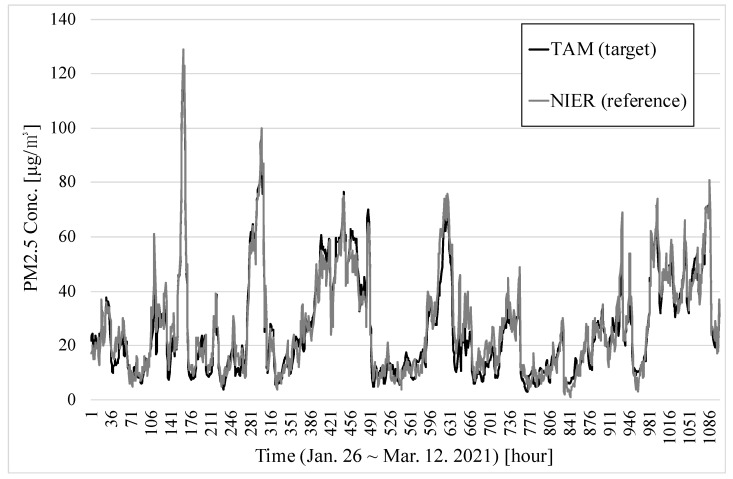
PM concentrations of TAM and the reference from 26 January to 9 March 2021.

Figure 10 illustrates the 1104 hourly average ${\mathrm{PM}}_{2.5}$ concentrations for TAM and the reference monitor for 46 days. The maximum value for TAM was 126 and the mean was 26.5; the maximum value for BAM was 129 and the mean was 27.5.

**Figure 11:**
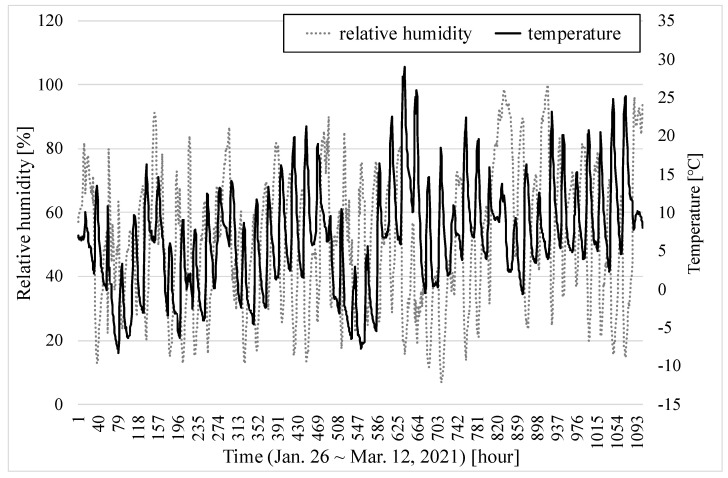
Temperature and relative humidity from 26 January to 9 March 2021.

Figure 11 also shows the temperature and relative humidity during the measurement period. The average temperature was 6.7 °C and the relative humidity ranged from 5.8% to 100%, being 50% on average.

**Figure 12:**
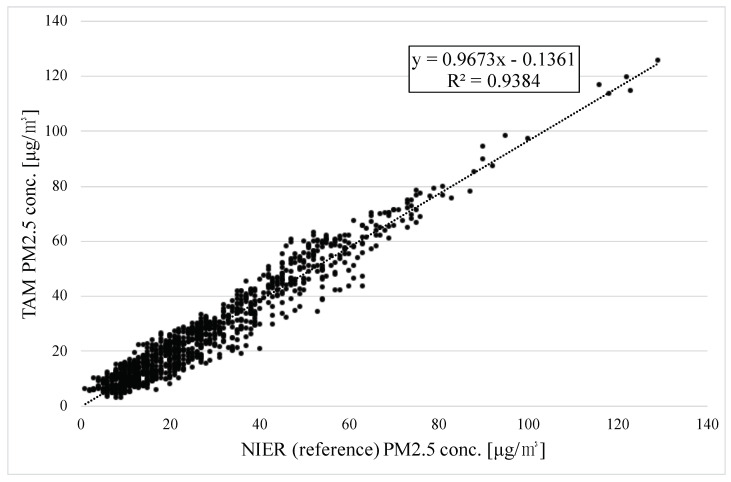
Correlation between TAM and NIER on hourly average from 26 January to 9 March 2021.

The correlation of 1104 hourly measurements is shown in Figure 12. The slope was 0.967, the intercept was −0.136, and $R^{2}$ was 0.938.

**Figure 13:**
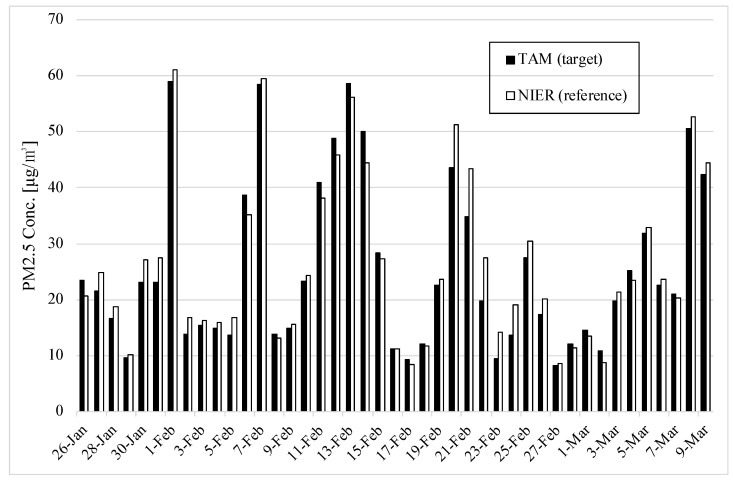
PM concentrations of TAM and NIER on daily average from 26 January to 9 March 2021.

The U.S. EPA evaluates candidate methods based on 46 daily average measurements. Thus, Figure 13 compares TAM and BAM in terms of daily average. The U.S. EPA states that class I should be used as a reference monitor, but in this experiment, a class II device, a monitor that is one level below class I, was used as a reference monitor.

**Figure 14:**
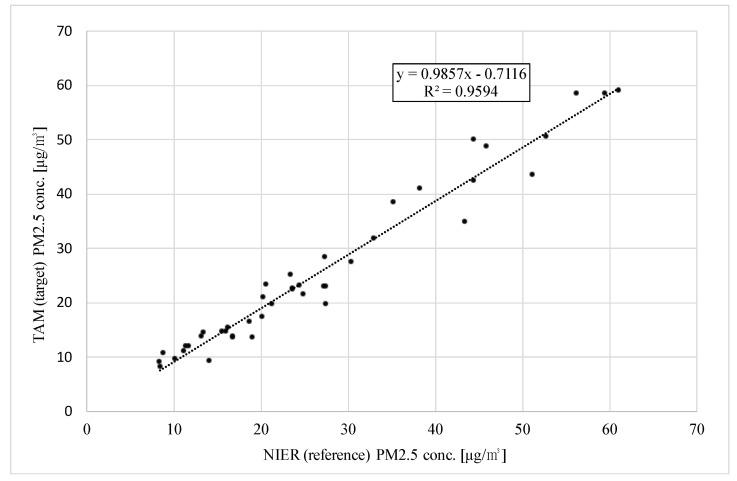
Correlation between TAM and NIER on daily average from 26 January to 9 March 2021.

Figure 14 shows the correlation graphs for 46 days of daily averages with slope, intercept, and $R^{2}$. The U.S. EPA chose the relative precision, slope, intercept, and correlation coefficient as evaluation indicators. Relative precision was previously addressed in Section 3.1. This section focuses on slope, intercept, and correlation coefficient.

Slope is defined by:

(6) $$\mathrm{slope}=\frac{\sum_{j=1}^{J}\left({\bar{R}}_{j}-\bar{R}\right)\left({\bar{C}}_{j}-\bar{C}\right)}{\sum_{j=1}^{J}{\left({\bar{R}}_{j}-\bar{R}\right)}^{2}},$$

where $R_{i,j}$ is the daily average concentration of the *i*th reference device on test day *j*, ${\bar{R}}_{j}$ is the mean concentration of all devices on day *j*, $\bar{R}$ is the average of ${\bar{R}}_{j}$ for all *j*, and *J* is the number of measurement days.

Intercept is defined by:

(7) $$\mathrm{intercept}=\bar{C}-\mathrm{slope}\times \bar{R}$$

To pass the U.S. EPA requirement, the slope must be between 0.9 and 1.10. The intercept must be between 15.05−(17.32×slope), but not less than −2.0, and 15.05−(13.20×slope), but not more than +2.0. Hence, for the slope of 0.986, the intercept must be between −2.0 and 2.0.

In the presented results, the slope was 0.986, and the intercept was −0.712. These results meet the requirement.

The U.S. EPA uses the Pearson correlation coefficient, r, which is not the coefficient of determination, $R^{2}$.

The correlation coefficient, *r*, is defined by:

(8) $$r=\frac{\sum_{j=1}^{J}\left({\bar{R}}_{j}-\bar{R}\right)\left({\bar{C}}_{j}-\bar{C}\right)}{\sqrt{\sum_{j=1}^{J}{\left({\bar{R}}_{j}-\bar{R}\right)}^{2}\sum_{j=1}^{J}{\left({\bar{C}}_{j}-\bar{C}\right)}^{2}}},$$

which must be greater than or equal to 0.93 if CCV ≤ 0.4, 0.85 + 0.2 × CCV if 0.4 ≤ CCV ≤ 0.5, or 0.95 if CCV ≥ 0.5.

CCV is defined as:

(9) $$\mathrm{CCV}=\frac{1}{\bar{R}}\sqrt{\frac{\sum_{j=1}^{J}{\left({\bar{R}}_{j}-\bar{R}\right)}^{2}}{J-1}}.$$

According to the results from this experiment, CCV was 0.55; so, *r* must be 0.95 or more per the U.S. EPA regulation. This experiment’s results confirmed that *r* was 0.979 ($R^{2}$ = 0.959).

Therefore, this experimental result shows that TAM satisfies the U.S. EPA indicators for relative precision, slope, intercept, and correlation coefficient.

This experiment was performed over 46 days in Korea, but the U.S. EPA class III certificate requires four test sites in the USA, three of which are performed for 23 days, respectively, and the other requires 46 days for winter and summer. The test sites are chosen to provide representative chemical and meteorological characteristics with respect to nitrates, sulfates, organic compounds, and various levels of temperature, humidity, wind, and elevation [29]. Hence, more stringent experiments remain to be performed on the proposed device.

## 4. Conclusions

The proposed OPM, TAM, incorporates TAC, which is equipped with a small and low-power dryer. TAC can make air flow with low energy because, naturally, air flows upwards, which is in the same direction as buoyancy caused by heat. TAM is not affected by wind according to Bernoulli’s principle because the inlet and the outlet are exposed in the same direction. The direct heating structure, which is small, allows the air to be quickly heated with low energy. The device consumes up to a total of 3.7 W, which is far less than a BAM system that consumes 520 W. The traditional methods manage the particle size before dehumidification, which can cause an error. However, TAM measures the size of particles after they pass through the dryer.

The indicators used to evaluate monitors include the relative precision, slope, intercept, and correlation coefficient. In the experimental results, the relative precision was 3.25%; the U.S. EPA stipulates that it must be 15% or less. The slope and the intercept were 0.986 and −0.712, respectively, which must be within [0.9, 1.1] and [−2.0, 2.0], respectively. The correlation coefficient was 0.979, which is above the regulated 0.95. The experimental results show that the presented monitor is a promising method for achieving U.S. EPA class III certification. In conclusion, the proposed OPM with an aerosol conditioner, TAM, is resistant to humidity, requires minimal maintenance, and ensures high measurement accuracy.

## Abbreviations

The following abbreviations are used in this manuscript:

PM	Particulate Matter
BAM	Beta-Radiation Attenuation Monitor
TEOM	Tapered Element Oscillating Microbalance
OPM	Optical Particle Monitor
RH	Relative Humidity
TAC	Tiny Aerosol Conditioner
TAM	TAC Inside Air Monitor
U.S. EPA	United Sates Environmental Protection Agency
NIER	National Institute of Environmental Research
